# PM2.5 Exposure Triggers Hypothalamic Oxidative and ER Stress Leading to Depressive-like Behaviors in Rats

**DOI:** 10.3390/ijms252413527

**Published:** 2024-12-17

**Authors:** Hi-Ju Kim, Ji-Hee Kim, Subo Lee, Phuong Anh Do, Ji Yong Lee, Seung-Kuy Cha, Jinhee Lee

**Affiliations:** 1 Department of Psychiatry, Yonsei University Wonju College of Medicine, Wonju 26426, Republic of Korea; sallypopo@hanmail.net; 2 Department of Occupational Therapy, Soonchunhyang University, Asan 31538, Republic of Korea; jhk1111@sch.ac.kr; 3 Department of Physiology, Yonsei University Wonju College of Medicine, Wonju 26426, Republic of Korea; sblee9605@naver.com (S.L.); phuonganh3110dkh@gmail.com (P.A.D.); 4 Department of Global Medical Science, Yonsei University Wonju College of Medicine, Wonju 26426, Republic of Korea; 5 Organelle Medicine Research Center, Yonsei University Wonju College of Medicine, Wonju 26426, Republic of Korea; 6 Research Institute of Hyperbaric Medicine and Science, Yonsei University Wonju College of Medicine, Wonju 26426, Republic of Korea; jylee740218@gmail.com

**Keywords:** ER stress, fine dust, hypothalamus, NOX4, oxidative stress

## Abstract

Epidemiological studies have linked fine dust pollution to depression, yet the underlying mechanisms remain unclear. Oxidative stress and endoplasmic reticulum (ER) stress are known contributors to depression, but their induction by particulate matter (PM), particularly PM2.5, in animal models has been limited. This study aimed to establish a rat model of PM2.5-induced depression-like behaviors and elucidate the underlying molecular mechanisms. Adult male Sprague–Dawley rats received daily intranasal PM2.5 for four weeks. Behavioral assessments, including the open field test (OFT), forced swim test (FST), and light-dark box (LDB) test, were conducted weekly. PM2.5-exposed rats displayed depressive-like behaviors, particularly in the FST, reflecting decreased motivation and learned helplessness. Molecular analyses indicated a specific increase in ER stress markers (CHOP, eIF2α, GRP78, and P16) and NOX4 in the hypothalamus, while other brain regions (striatum, cortex, and hippocampus) were not as pronounced. Additionally, PM2.5 exposure reduced tyrosine hydroxylase (TH) levels in the hypothalamus, suggesting impaired dopamine synthesis. These findings indicate that PM2.5 induces depressive-like behaviors via hypothalamic ER stress and oxidative stress pathways, leading to dopaminergic dysfunction. Targeting oxidative and ER stress within the hypothalamus may offer new therapeutic strategies for treating depression associated with environmental pollutants.

## 1. Introduction

The hallmarks of major depressive disorder (MDD) are fatigue, prolonged sadness, reduced energy, and reduced interest in activities for at least two weeks. MDD is the most common mental health disorder globally and a leading cause of disability [1,2]. Recent studies have increasingly linked air pollution exposure with various mental health issues, including depression [3,4,5,6]. Time-series analyses have shown associations between air pollution and increased emergency department visits for depressive symptoms [7]. The influence of air quality on vulnerable groups, such as older persons, has been highlighted by previous research that primarily identifies a relationship between air pollution and mental health in elderly populations. Exposure to PM10, NO_2_, and O_3_ has been linked to emotional symptoms [8]. Moreover, meta-analyses reinforced the association between elevated ambient PM levels and increased risk for depression and suicide [9]. Long-term exposure to PM2.5 has been linked in studies to an increased chance of developing MDD, especially in individuals who already have chronic illnesses that make their conditions more susceptible to its effects [10].

Both epidemiological and experimental studies suggest that air pollution exposure contributes to central nervous system (CNS) disorders, cognitive impairments, neuroinflammation, and neurological abnormalities in humans and animals [11,12]. Although the precise mechanisms underlying the neurotoxic effects of airborne PM remain unclear, emerging evidence suggests that endoplasmic reticulum (ER) stress and oxidative stress play important roles in driving functional and structural dysfunctions in the CNS associated with mental health disorders [13,14]. Exposure to fine dust, including PM2.5, has been shown to cause ER stress and oxidative stress, which are associated with mental health problems [15,16,17,18]. However, animal studies have demonstrated limited induction of this stress response by fine dust pollution, and the specific brain regions affected remain unclear.

Emerging evidence suggests additional links between ER stress and depression, including the generation of reactive oxygen species (ROS), Ca^2+^ release from the ER, and initiation of acute phase responses [15,16,17]. The brain’s high oxygen consumption and limited antioxidant defenses make it particularly vulnerable to oxidative damage, strongly implicated in neurodegeneration and MDD development [18]. Depressed patients exhibit reduced serum levels of antioxidants and elevated levels of free radicals and oxidative damage markers compared to controls [19]. Elevated levels of oxidative stress-induced damages, such as lipid peroxidation, DNA damage, and protein oxidation, have been observed in both postmortem brain tissue from individuals with depression and in animal models exacerbating neuronal damage and dysfunction [20,21,22]. Multiple brain cells, such as neurons, microglia, and astrocytes, can produce ROS that alters synaptic plasticity and potentially contributes to depressive behaviors [23,24,25]. NADPH oxidase (NOX) enzymes, particularly NOX4, are present in different brain cell types, and ROS generation via NOX-dependent mechanisms significantly contributes to depression development [26,27,28,29,30].

This study used a rodent intranasal administration model to address the translational gap between animal models and human conditions, mimicking real-world PM2.5 exposure pathways. This aligns with prior evidence showing PM’s translocation to the brain via the olfactory nerve [31,32].

Anhedonia, a core feature of MDD, is associated with deficits in hedonic response, primarily influenced by the dopamine system, which is one of the main pathways to regulate motivation, feelings of helplessness, and enjoyment that are hallmarks of depression [33]. Different brain regions, such as the hippocampus and hypothalamus, are critical in regulating dopaminergic activity, suggesting the intricate role of dopamine in mood regulation [33]. However, limited research has examined how fine dust exposure triggers depressive behaviors, particularly in specific neurologic pathophysiology, brain regions, and molecular pathways. Given the limited evidence of oxidative and ER stress induction by PM2.5 in previous animal models, it is crucial to establish a model that more effectively simulates the human condition.

The present study aims to elucidate the mechanisms by which PM2.5 exposure contributes to the development of depression. Specifically, we investigate whether intranasal administration of PM2.5 induces depressive-like behaviors in rats, as assessed through established behavioral tests, including the light-dark box (LDB) test, forced swim test (FST), and open field test (OFT). Furthermore, we examine the potential association between these behavioral changes, increased ER stress, and oxidative stress markers in specific brain regions.

## 2. Results

### 2.1. PM2.5 Induces ER Stress and Alters Neuronal Injury Markers in the Hypothalamus

**Figure 1:**
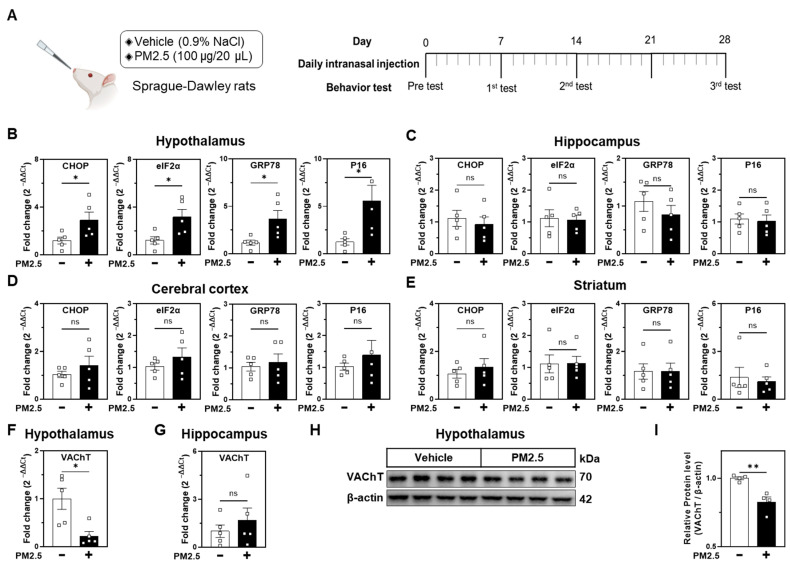
Intranasal PM2.5 exposure induces ER stress, cell senescence, and reduced neuronal markers in specific rat brain regions. (**A**) Experimental timeline illustrating daily intranasal injections of either vehicle (0.9% NaCl) or PM2.5 (100 μg/20 μL) administered to Sprague–Dawley rats for 28 days, followed by behavioral tests. Vehicle, *n* = 5. PM2.5, *n* = 5. (**B**–**E**) qPCR analysis showing increased expression of ER stress and cell senescence markers (CHOP, eIF2α, GRP78, and P16) in the hypothalamus (B), hippocampus (C), cortex (D), and striatum (E) following PM2.5 exposure, with significant changes observed in the hypothalamus, cortex, and striatum. (**F**,**G**) PM2.5 exposure reduced mRNA expression of VAChT in the hypothalamus (F) but not in the hippocampus (G). (**H**) A representative immunoblot showing decreased VAChT protein level in the hypothalamus following PM2.5 treatment. β-actin served as a loading control. (**I**) Quantification of VAChT protein expression from panel H. Data are presented as mean ± SEM, with comparisons made using Student’s *t*-test in panels (**B**–**G**,**I**). ns., not significant. * *p* ≤ 0.05, ** *p* ≤ 0.01 vs. Vehicle, *n* = 5.

We employed a nasal injection model of PM2.5 in rats to simulate air pollution exposure. Figure 1A outlines the experimental timeline, which includes daily nasal PM2.5 (100 μg/20 μL) or vehicle injections, followed by behavioral assessments (OFT, FST, and LDB). Seven-week-old male Sprague–Dawley rats (250 g) underwent 28 days of treatment before brain dissection to analyze ER stress, cell senescence, and neuronal injury markers.

Quantitative PCR analysis indicated that PM2.5 significantly increased ER stress markers (CHOP, eIF2α, and GRP78) and the cellular senescence marker P16 in the hypothalamus (Figure 1B), while no significant changes were detected in the hippocampus, cerebral cortex, and striatum (Figure 1C–E). These findings suggest region-specific effects of PM2.5, with a pronounced impact on the hypothalamus.

Additionally, we evaluated the expression of vesicular acetylcholine transporter (VAChT) as neuronal markers. PM2.5 exposure significantly reduced mRNA expression of VAChT in the hypothalamus, with no such changes in the hippocampus (Figure 1F,G). In line with this, VAChT protein levels were also reduced in the hypothalamus (Figure 1H,I). This reduction in VAChT, a critical cholinergic marker [34], suggests that PM2.5 increases ER stress and cellular senescence and may also impair cholinergic signaling in the hypothalamus. These results demonstrate that the hypothalamus is a vulnerable brain region to intranasally administered PM2.5-induced neurotoxicity.

### 2.2. PM2.5 Increases NOX4 Expression in the Hypothalamus

**Figure 2:**
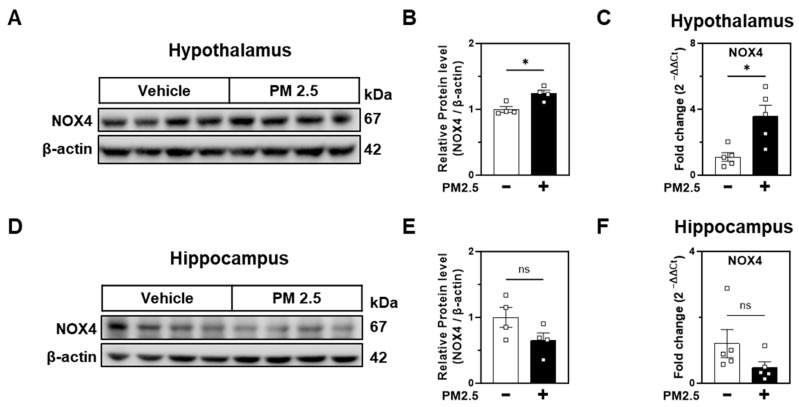
PM2.5 nasal administration increases protein and mRNA levels of NOX4 in the hypothalamus but not in the hippocampus. (**A**,**D**) Western blot analysis of NOX4 protein levels in the hypothalamus (**A**) and hippocampus (**D**) following vehicle or PM2.5 treatment. β-actin served as a loading control. (**B**,**E**) Quantification of NOX4 protein levels from panels A and D, respectively. (**C**,**F**) Real-time qPCR for NOX4 mRNA level in the (**C**) hypothalamus and (**F**) hippocampus following vehicle or PM2.5 treatment. Data are presented as mean ± SEM, with comparisons made using Student’s *t*-test in panels (**B**,**D**). ns., not significant. * *p* ≤ 0.05 vs. Vehicle, *n* = 4.

Given our previous findings on NOX4-driven oxidative stress as a mechanism of PM2.5 neurotoxicity in neuronal cells [35], we examined NOX4 expression in both the hypothalamus and hippocampus. NOX4 protein was significantly increased in the hypothalamus of PM2.5-exposed rats compared to the vehicle group (Figure 2A,B). In contrast, no noticeable difference was observed in the hippocampus (Figure 2D,E). Following protein data, qPCR analysis showed increased NOX4 mRNA in the hypothalamus after PM2.5 exposure but not in the hippocampus (Figure 2C,F). This increased NOX 4 expression indicates enhanced oxidative stress in the hypothalamus [26,27,28,29,30]. These findings underscore a region-specific induction of oxidative stress by PM2.5, characterized by increased NOX4 expression in the hypothalamus.

### 2.3. PM2.5 Triggers ER Stress, Mitochondrial ROS Production, and Neuronal Injury in Neuro-2A

**Figure 3:**
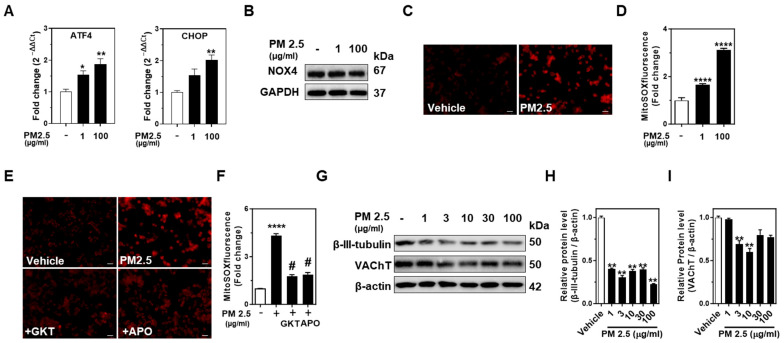
PM2.5 induces ER stress and mitochondrial ROS production, reducing neuronal markers in Neuro-2A cells. (**A**) q-PCR analysis of ER stress markers ATF4 and CHOP in Neuro-2A cells treated with low (1 μg/mL) and high (100 μg/mL) doses of PM2.5. (**B**) Western blot analysis of NOX4 protein levels in Neuro-2A cells treated with vehicle, 1 μg/mL, and 100 μg/mL of PM2.5, with β-actin as a loading control. (**C**,**D**) Mitochondrial ROS production in Neuro-2A cells treated with PM2.5 (100 μg/mL), showing a significant increase in ROS levels compared to vehicle control. (**E**,**F**) PM2.5-induced mitochondrial ROS increase was significantly reduced by NOX inhibitors GKT137831 and Apocynin. (**G**) Western blot analysis of β-III-tubulin and VAChT protein levels in Neuro-2A cells treated with increasing concentrations of PM2.5 (1, 3, 10, 30, and 100 μg/mL), with β-actin as a loading control. (**H**,**I**) Quantifying β-III-tubulin (**H**) and VAChT (**I**) protein levels from panel G, respectively. Scale bars, 5 μm. Bar graphs are expressed as mean ± SEM and analyzed using one-way ANOVA (**A**,**D**,**F**,**H**,**I**). * *p* ≤ 0.05, ** *p* ≤ 0.01, **** *p* ≤ 0.001 vs. Vehicle. # *p* ≤ 0.05 vs. PM2.5.

In Neuro-2A cells, PM2.5 exposure at both low (1 μg/mL) and high (100 μg/mL) doses led to a dose-dependent increase in ER stress markers (ATF4 and CHOP) at the mRNA level, as shown by q-PCR analysis (Figure 3A). Our previous work identified NOX4 as the main NOX isoform responsible for mitochondria-derived ROS generation in Neuro-2A cells [35]. Following PM2.5 treatment, NOX4 protein levels remained unchanged (Figure 3B), supporting the notion that while PM2.5 induces ER stress, it does not directly alter NOX4 protein expression in these cells. Mitochondrial ROS production, assessed via mitoSOX staining, significantly increased dose-dependent manner, even at the low PM2.5 exposure (Figure 3C,D). Notably, mitochondrial ROS levels at 100 μg/mL PM2.5 were reduced considerably by the NOX inhibitors GKT137831 and Apocynin, suggesting a role for NOX activity in PM2.5-induced ROS generation (Figure 3E,F). Western blot analysis further revealed that PM2.5 exposure reduced β-III-tubulin and VAChT protein levels across various concentrations (1, 3, 10, 30, and 100 μg/mL), with a notable decrease in β-III-tubulin starting at 1 μg/mL.

VAChT levels declined significantly at 3 and 10 μg/mL, with a trend toward further reduction at higher concentrations, though not statistically significant (Figure 3G–I). These findings demonstrate that PM2.5 induces ER and mitochondrial stress, promoting neuronal injury in Neuro-2A cells.

### 2.4. Nasal PM2.5 Exposure Induces Depressive Behavior and Reduces TH Expression in the Hypothalamus

**Figure 4:**
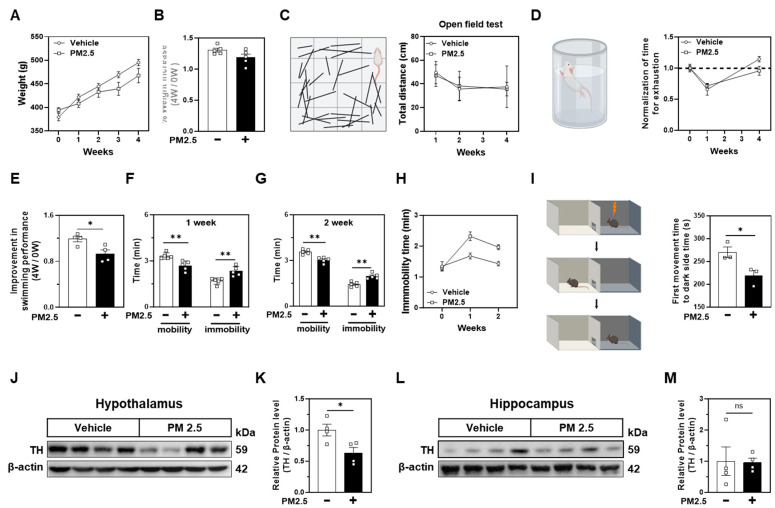
Nasal administration of PM2.5 induces depressive-like behavior and reduces TH expression in rats. (**A**) Weight tracking over 4 weeks for Vehicle (*n* = 5) and PM2.5-treated rats (*n* = 5). (**B**) Percent weight increase calculated by dividing the weight at 4 weeks by the initial weight at 0 weeks. (**C**) Open field test (left). Total distance traveled by vehicle- and PM2.5-treated rats at 1, 2, and 4 weeks (right). (**D**) Forced swim test (left). Time until exhaustion in vehicle- and PM2.5-treated rats, normalized to week 0, and compared across 1 and 4 weeks. (**E**) Swimming improvement over 4 weeks, calculated by dividing the weight at 4 weeks by the weight at 0 weeks. (**F**,**G**) Mobility and immobility times were recorded during the forced swim test for 5 min at 1 (**F**) and 2 weeks (**G**) post-treatment. (**H**) Immobility time comparison during the forced swim test over weeks 0, 1, and 2. (**I**) Dark-light box test with electric shock (left). The time to move to the dark side after the shock was recorded to assess short-term memory (right). (**J**,**L**) Western blot analysis of tyrosine hydroxylase (TH) protein levels in the (**J**) hypothalamus or (**L**) hippocampus of vehicle- and PM2.5-treated rats, with β-actin as a loading control. (**K**,**M**) Quantification of TH protein levels normalized to β-actin in (**K**) the hypothalamus and (**M**) the hippocampus, respectively. Data are presented as mean ± SEM, and analyzed with Student’s *t*-test (**B**,**E**,**F**,**G**,**I**,**K**,**M**). ns., not significant. * *p* ≤ 0.05, ** *p* ≤ 0.01 vs. Vehicle.

To examine the behavioral effects of PM2.5, we conducted four-week animal behavior experiments alongside daily nasal PM2.5 administration. PM2.5-exposed rats exhibited less weight gain than vehicle controls (Figure 4A), though this trend was not statistically significant (Figure 4B), suggesting potential effects on metabolism or appetite. The total distance traveled by the groups in the open field test (OFT) did not differ significantly, suggesting that PM2.5 did not affect locomotor activity (Figure 4C). In the forced swim test (FST), PM2.5-exposed rats displayed reduced exhaustion and swim improvement times (Figure 4D,E), indicating an increased vulnerability to stress. Mobility analysis revealed decreased mobility and increased immobility in PM2.5-injected rats during the first two weeks of FST (Figure 4F–H), supporting a depressive-like behavioral profile. In the light-dark box (LDB) test, PM2.5-injected rats spent less time moving to the dark compartment following an electric shock, suggesting impaired memory and heightened anxiety-like behavior (Figure 4I).

A tyrosine hydroxylase (TH) expression in the hypothalamus was significantly decreased in PM2.5-treated rats (Figure 4J,K), suggesting reduced dopamine synthesis linked to depressive behaviors. Conversely, TH expression remained unchanged in the hippocampus (Figure 4L,M), indicating a region-specific impact of PM2.5 on dopaminergic pathways [36,37,38].

Together, these results suggest that nasal PM2.5 exposure induces depressive-like behaviors through suppression of hypothalamic TH, potentially leading to diminished dopamine synthesis and contributing to mechanisms underlying PM2.5’s impact on major depressive disorder.

## 3. Discussion

This study demonstrates that PM2.5 exposure induces depressive-like behaviors in rats, primarily targeting hypothalamic dysfunction. Results reveal a significant increase in oxidative and ER stress markers, specifically NOX4, CHOP, eIF2α, and GRP78, in the hypothalamus, while no such changes were observed in other brain regions. These brain region-specific features indicate that the hypothalamus is a critical area in the neurotoxic response to PM2.5 exposure, aligning with its established role in mood regulation, stress response, and energy balance and underscoring its heightened sensitivity to environmental pollutants like PM2.5 [39,40,41]. Oxidative and ER stress are implicated in depressive behaviors; these findings support the role of cellular stress response in the neurotoxic effects of air pollution [14,42,43]. In addition to the markers evaluated in this study, previous research highlights the significance of other ER stress markers, such as ATF6, XBP1, and IRE1α, in neurotoxic responses to environmental pollutants like PM2.5 [44,45]. Incorporating these markers in future studies could provide a more comprehensive understanding of the unfolded protein response pathways involved in PM2.5-induced cellular stress.

In addition to the stress markers, PM2.5 exposure significantly reduced the expression of vesicular acetylcholine transporter (VAChT) and β-III-tubulin in the hypothalamus at both the mRNA and protein levels. VAChT and β-III-tubulin are crucial for regulating cholinergic neurotransmission and maintaining neuronal structure and neurogenesis. The reduction in VAChT suggests impaired cholinergic signaling, strongly linked to mood regulation and cognitive function [34]. This disruption in cholinergic pathways likely contributes to the depressive-like behaviors observed in our study, such as increased immobility in the FST and impaired memory in the LDB test. These findings align with studies linking cholinergic dysfunction to both depression and cognitive decline, further connecting PM2.5 exposure to neurodegenerative processes [46,47].

Reduced expression of β-III-tubulin in the hypothalamus caused by PM2.5 suggests that PM2.5 may influence neuronal plasticity and neurogenesis, resulting in structural and functional alterations linked to depressive behaviors. Neurodegenerative disorders and depression are associated with abnormalities in neuronal plasticity and structural integrity, which are essential for mood regulation and cognitive function [34,48]. This decrease lends more credence to the idea that acute stress responses and long-term alterations in neuronal health and plasticity are involved in PM2.5-induced neurotoxicity, which may result in long-lasting cognitive and behavioral impairments.

Behaviorally, rats exposed to PM2.5 exhibited classic signs of depression, including reduced motivation and increased helplessness, as evidenced by the forced swim test. These findings align with the dopaminergic dysfunction observed in this study, indicated by reduced TH expression, a key enzyme in dopamine synthesis. Dopamine plays a central role in motivation, reward, and pleasure, and reduced dopaminergic activity is a well-known feature of depression, particularly in relation to anhedonia and psychomotor retardation [33,49]. The observed decrease in TH expression in the hypothalamus suggests impaired dopamine synthesis, contributing to the helplessness and decreased motivation observed in this study. These findings are in line with other depression models in which disrupted hypothalamic dopaminergic signaling leads to reduced motivation and impaired stress coping [49,50].

Furthermore, the light-dark box (LDB) test revealed impaired memory and anxiety-like behaviors in PM2.5-treated rats. These cognitive impairments are consistent with previous findings linking air pollution exposure to cognitive dysfunction and neurodegenerative changes. Memory impairment, a common symptom of depression, may result from the combined effects of increased ER stress and oxidative stress, cholinergic dysfunction, and impaired neuroplasticity, as indicated by the elevation of ER stress markers and NOX4, and reductions in VAChT and β-III-tubulin [48,51]. These cognitive deficits further support that PM2.5 exposure not only affects mood but also contributes to broader neurological dysfunction, with potential long-term consequences for brain health.

The concurrent reduction in VAChT and β-III-tubulin expression, along with the increase in ER and oxidative stress markers, suggests a dual mechanism by which PM2.5 impacts brain function: through both direct cellular damage via ER stress and mitochondrial oxidative stress, and indirect effects on neurotransmission and neuroplasticity. The hypothalamus is a susceptible region, making it an important area of focus for future research on environmental contaminants and mental health.

Although rodent models offer significant insights into the mechanistic impacts of PM2.5 on the CNS, they may only partially emulate the intricacies of human illness. Divergence in brain networks, neurochemical systems, and environmental interactions between rodents and humans may affect the translational relevance of these findings. Our study underlines the significance of the hypothalamus as a principal target of PM2.5-induced neurotoxicity.

Notably, this study highlights the hypothalamus as a primary target of PM2.5-induced neurotoxicity. Multiple studies have demonstrated that PM2.5 can also affect other brain areas linking neuroplasticity and cognitive function, including the hippocampus [45] and cortex [52]. Future studies exploring these regions, along with systemic pathways such as inflammation and metabolic interactions, will provide a more comprehensive understanding of the neurotoxic effects of PM2.5.

Our findings indicate that chronic exposure to higher levels of PM2.5 may hinder dopamine synthesis, potentially leading to mood dysregulation and increased vulnerability to stress-related diseases. Multiple preventive methods of reducing air pollutants, such as using air purifiers, evasion of high-pollution zones, and incorporating antioxidant-rich meals, may assist in lowering individual exposure and its consequences. On a larger scale, public health interventions, including more stringent air quality standards, promoting renewable energy sources, and managing industrial emissions, are essential for decreasing PM2.5 levels.

In conclusion, our findings provide compelling evidence that PM2.5 exposure induces depressive-like behaviors through a combination of ER and mitochondrial ROS stress, causing cholinergic and dopaminergic dysfunction predominantly in the hypothalamus. PM2.5-induced reductions in neuronal markers VAChT and β-III-tubulin suggest that air pollution impairs mood, cognitive function, and neuroplasticity. These results underscore the critical need to mitigate air pollution exposure to protect mental health and highlight potential therapeutic targets for pollution-induced depression, particularly pathways involving ER and oxidative stress linked to cholinergic and dopaminergic signaling.

## 4. Materials and Methods

### 4.1. Cell Culture

The Neuro-2A (CCL-131) murine neuroblastoma cell line was obtained from the American Type Culture Collection (ATCC). Cells were maintained in Eagle’s Minimum Essential Medium (EMEM) (#30-2003, ATCC, Manassas, VA, USA), supplemented with 10% fetal bovine serum (FBS) (#16000-044, Gibco, Grand Island, NY, USA) and 1% penicillin/streptomycin solution (#SV30010, HyClone, Logan, UT, USA). Cultures were incubated at 37 °C in a humidified atmosphere containing 5% CO_2_.

### 4.2. Animal

All animal experiments and surgical procedures were approved by the Institutional Animal Care and Use Committee at Yonsei University Wonju College of Medicine (Identification code: YWC-210615-1), and procedures were conducted according to the National Institutes of Health Guidelines for the Care and Use of Laboratory Animals. Adult male Sprague–Dawley rats (250 g) were obtained from Dae Han Bio Link (Eumseong, Republic of Korea) and housed under a 12 h light/12 h dark cycle at a constant room temperature (20–22 °C), with free access to food and water.

### 4.3. Intranasal Injection

Sprague–Dawley male rats (8 weeks old; body weight, 250–300 g; *n* = 15) were used. Five rats were assigned to each of the three groups: Control, Vehicle (0.9% NaCl injected), and PM2.5 (100 μg/20 μL). Vehicle and PM2.5 groups received intranasal injections of 0.9% NaCl or PM2.5 (100 μg/20 μL), respectively, once daily for 1 month (28 days).

### 4.4. Weight Tracking

All rats were weighed weekly. Weight increases were calculated as the weight at the last week (4th week) divided by the first week (0 weeks before injection).

### 4.5. Open Field Test (OFT)

The open field used consisted of a square arena made of plastic board. Each rat from the three groups (control, vehicle, and PM) was placed in the box for 10 min. Their behavior in the box was video recorded. The total duration each rat spent in the center zone and the distance traveled in the 10-min observation period were analyzed automatically using SMART^®^ (PanLab, S.L., Barcelona, Spain). Once the test had been completed for each rat, the test arena was cleaned and wiped with 70% ethanol, followed by wiping with a dry paper towel to ensure the test field was clean and dry. Each group contained five rats.

### 4.6. Forced Swim Test (FST)

All rats performed a forced swimming test three times: before injection, and then after starting the injection during the 1st week, 2nd week, and 4th week. This test was recorded for 5 min and measured the time from the start of swimming to exhaustion. Swim improvement was calculated as the ratio of the last week (4th week) to the first week (0 weeks before injection). Mobility time was measured by the time spent climbing and scrambling, and immobility time was recorded as the time spent not moving.

### 4.7. Light-Dark Box (LDB)

Light/dark box consisted of a lighted safe side and a dark, shock side separated by a trap door. During the training phase, rats were placed on the safe side of the box, facing a corner opposite the door for 10 min. After that, the trap door was opened, and rats could enter the dark box. The latency before entering the novel dark box was measured. Four seconds after the animals entered the dark side, we closed the door and applied a scrambled electrical foot shock through electrified steel rods in the floor of the box. The rats were kept in the dark compartment for 10 s before returning to their home cage. In the test phase, the rats were placed on the lighted side, and the latency before entering the dark box was measured. Each group contained 5 rats (male).

### 4.8. Western Blot

Western blotting (WB) was performed as described previously [35]. Sacrificed rat tissues were lysed using TissueLyser with beads in RIPA, including 1% protease inhibitor cocktails and 1% phosphatase inhibitor cocktails. The primary antibodies used for immunoblotting were: GAPDH (diluted 1:2500, sc-365062) and TH (diluted 1:1000, sc-25269) from Santa Cruz Biotechnology (Santa Cruz, CA, USA); VAChT (diluted 1:1000, #75-020) from NeuroMab Facility (UC Davis/NIH, CA, USA); β-actin (diluted 1:2500, ab6276) and β-III-tubulin (#ab7751, diluted 1:1000) from Abcam (Cambridge, UK); NOX4 (diluted 1:1000, NB110-58849) from Novus Biologicals (Littleton, CO, USA). Bands in the immunoblotting were detected and quantified using a ChemiDoc XRS+ System (https://www.bio-rad.com/ko-kr/product/chemidoc-xrs-system?ID=NINJHRKG4 (accessed on 14 December 2024), Bio-Rad, Hercules, CA, USA) and Image Lab Software 6.0.1 (Bio-Rad), respectively.

### 4.9. Real-Time Polymerase Chain Reaction (q-PCR)

RNA preparation, complementary DNA (cDNA) synthesis, and q-PCR were performed as previously described [35]. Real-time PCR amplification was carried out using a QuantStudio 6 Flex Real-Time PCR System (Thermo Fisher, Waltham, MA, USA) and the QuantiTect SYBR Green PCR kit (Qiagen, Hilden, Germany). The thermal cycling conditions were as follows: an initial polymerase activation for 15 min at 95 °C, followed by 40 cycles of 15 s at 95 °C for denaturation and 60 s at 60 °C for annealing and extension. Primer sequences for all genes in this study are listed in Supplementary Table S1. Gene expression was calculated using the 2^−ΔΔCt^ method, with 18 s as the reference gene.

### 4.10. Measurement of Mitochondrial ROS

Cells were loaded with 5 μM MitoSOX (#M36008, Molecular Probes, Invitrogen, Waltham, MA, USA) for 10 min in warmed PBS at 37 °C and then washed with warm PBS [35]. GKT137831 (5 μM) and Apocynin (10 μM) were added 10 min before a 1-h co-incubation with fine dust (PM2.5; 1 or 100 μg/mL). MitoSOX fluorescence images were captured within 5 min using excitation at 510 nm and emission at 580 nm (IX-81; Olympus, Tokyo, Japan) with a confocal spinning disk (CSU10; Yokogawa, Tokyo, Japan). Data from more than 10 images from three independent experiments were averaged after background fluorescence correction using MetaMorph 6.3 software (Molecular Devices, San Jose, CA, USA).

### 4.11. Statistics

Data were analyzed using the GraphPad Prism 8 software program (GraphPad Software, San Diego, CA, USA). Statistical comparisons between two data groups were performed using a *t*-test (and nonparametric tests) in column analyses. *p*-values ≤ 0.05 were considered statistically significant, and data are presented as mean ± S.E.M.

## 5. Conclusions

Our study demonstrates that PM2.5 exposure induces depressive-like behaviors in rats by selectively impacting the hypothalamus, leading to increased ER and oxidative stress. This exposure also significantly reduced the expression of tyrosine hydroxylase, VAChT, and β-III-tubulin. These changes indicate disruptions in dopaminergic and cholinergic signaling and alterations in neuronal markers. These alterations likely contribute to core depressive symptoms such as helplessness, anhedonia, and memory impairments. Our findings highlight the unique vulnerability of the hypothalamus to environmental pollutants like PM2.5 and suggest that targeting ER and oxidative stress and neuronal injury pathways may offer therapeutic potential for mitigating pollution-induced depression. Reducing air pollution is essential for safeguarding mental health and preventing its neurotoxic effects.

## Data Availability

The data used and/or analyzed during the current study are available from the corresponding author upon reasonable request.

## Supplementary Materials

The supporting information can be downloaded at: https://www.mdpi.com/article/10.3390/ijms252413527/s1.
