# Supplementary material for: PM2.5 Exposure Triggers Hypothalamic Oxidative and ER Stress Leading to Depressive-like Behaviors in Rats
Source: Int J Mol Sci. 2024 Dec 17;25(24):13527. doi: 10.3390/ijms252413527 (PMC11677780; doi:10.3390/ijms252413527)
Supplement: Supplementary file 1 [file ijms-25-13527-s001.zip › ijms-3352515-supplementary.pdf]

Supplementary Table S1. List of primer sequences used for PCR

Gene	Species	Primer sequence (5′ to 3′)	
18s	H, M, R	Forward	AACCCGTTGAACCCCATT
		Reverse	CCATCCAATCGGTAGTAGCG
VAChT	R	Forward	GCCACATCGTTCACTCTCTTG
		Reverse	CGGTTCATCAAGCAACACATC
CHOP	R	Forward	CCAGCAGAGGTCACAAGCAC
		Reverse	CGCACTGACCACTCTGTTTC
	M	Forward	CTGGAAGCCTGGTATGAGGAT
		Reverse	CAGGGTCAAGAGTAGTGAAGGT
eIF2α	R	Forward	TTCGACCTCCCAAAAGCAGT
		Reverse	TTGTAGGTTAGGCGTCCCAG
GRP78	R	Forward	CAGATCTTCTCCACGGCTTC
		Reverse	GCAGGAGGAATTCCAGTCAG
p16	R	Forward	TGCAGATAGACTAGCCAGGGC
		Reverse	CTCGCAGTTCGAATCTGCAC
TH	R	Forward	GCCCCCACCTGGAGTATTTTG
		Reverse	AGACACCCGACGCACAGAGC
AFT	M	Forward	GCTCTTGACCACGTTGGATG
		Reverse	GGCCAATTGGGTTCACTGTC
